# Graphic medicine in academic health science library collections

**DOI:** 10.5195/jmla.2025.1962

**Published:** 2025-07-01

**Authors:** Janet Chan, Melinda H. Berg, Krystal Bullers, Tsu Yin Lue

**Affiliations:** 1 janetchan@usf.edu, Research and Education Librarian, USF Health Libraries, University of South Florida, Tampa, FL; 2 mberg4@usf.edu, Cataloging and Metadata Librarian, USF Health Libraries, University of South Florida, Tampa, FL; 3 krystalb@usf.edu, Research and Education Librarian, USF Health Libraries, University of South Florida, Tampa, FL; 4 tlue2@usf.edu, Research and Education Staff Assistant, USF Health Libraries, University of South Florida, Tampa, FL

**Keywords:** Graphic medicine, collection development, health science libraries

## Abstract

**Objective:**

Academic health science library catalogs were analyzed to determine the presence and frequency of graphic medicine titles in print format in the collections. The secondary objectives were to gauge if students could access graphic medicine titles, through other libraries within the same system or as eBooks, and to examine if libraries highlighted graphic medicine collections and their uses on their websites.

**Methods:**

A convenience sample of health science libraries was created from the Association of Academic Health Science Libraries member list. A title list was developed from collection resources and award lists for graphic medicine and graphic novels. Data was collected from public-facing library catalogs.

**Results:**

Fifty-six percent of the libraries analyzed had at least one title from the list in their collections available as print. An additional thirty percent had at least one title available as an eBook, leaving only fourteen percent with no graphic medicine titles.

**Conclusions:**

This study provides a starting point in describing the prevalence and breadth of graphic medicine collections in academic health science libraries. Although their presence may be small, our findings suggest that graphic medicine is being collected by academic health science libraries. Academic librarians can support the growing interest in the comic art format by incorporating graphic medicine into their collections and educating their patrons on this important genre.

## INTRODUCTION

Biology, physiology, and biochemistry may be the foundation of medical education, but people are more than just a disease state and medical decision-making is not always straight forward. To bridge this gap, medical school curricula began incorporating medical humanities in the 1960s as a way to integrate ethical considerations and understanding of the patient's experience into the harder edges of medical practice [1]. By 2018, ninety-four percent of medical schools participating in the Liaison Committee on Medical Education (LCME) medical school questionnaire reported medical humanities as a part of required or elective courses [2]. The integration of humanities into medical curriculum has also been determined as foundational content for physician development by the Association of American Medical Colleges [3].

The expansion of the humanities within medical school curricula has been accompanied by an increase in the subject areas that comprise medical humanities to include narrative medicine, visual arts, theater, film, music, and comics [4]. Comics specifically have evolved from their early use as medical caricatures [5] into the unique field of study known as graphic medicine [6]. The term “graphic medicine” was first coined by comic artist and physician, Ian Williams, in 2007 as “the intersection between the medium of comics and the discourse of healthcare” [6,7]. This definition illustrates the breadth of graphic medicine across health disciplines with an active presence in fields such as nursing, public health, and bioethics [8].

Although comics may not intuitively seem appropriate for the serious nature of health care professions, the addition of visual elements in comics can help impart understanding and convey experiences more effectively than text alone [9]. For example, in the public health arena, Meredith Li-Vollmer [10] uses comics to communicate how global issues such as climate change impact local communities.

**Figure 1:**
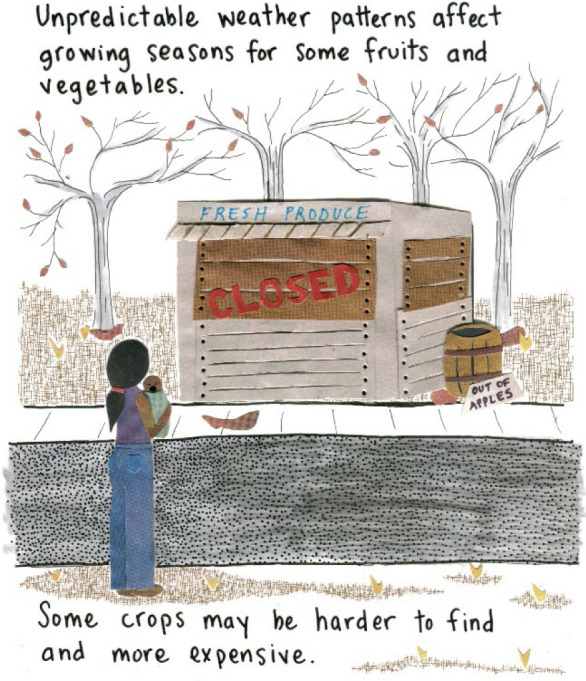
Climate Changes Panel 1. Courtesy of Public Health – Seattle & King County. Written by Meredith Li-Vollmer, artwork by Mita Mahato.

In Figure 1, the text explains that the produce supply is changing due to altered weather patterns. The illustration shows a woman and child at a closed food stand with an empty apple basket drawing attention to how global change translates on a local level. Comic art connects these global issues and their local impacts, fostering a deeper understanding of how these issues may affect communities on a personal level. This unique quality is also what makes comics appropriate for promoting understanding of difficult subjects in health science education. Graphic medicine can also be an effective tool in clinical care by providing a connection between hard to grasp concepts about the body and how they affect specific individuals.

In medical education, graphic medicine's versatility is evident – from illustrating specific concepts in a single assignment to forming the foundation for an entire course. For example, Masel et al [11] assigned medical students to read one of three comic art panels illustrating difficult situations experienced by either patients or doctors and then write a reflection on the situation depicted in the comics. This application demonstrated how a single comic art panel can promote reflection on a complex situation. Similarly, studies conducted by Green [12] and by Ronan and Czerwiec [13] describe implementing a four-week course of reading and creating comics to promote empathy and understanding among medical students of the patient experience across a variety of disease states.

While promoting empathy and understanding of the patient's experience remains a significant contribution of graphic medicine, its evolution extends beyond this aspect. Since its initial description in 2010 [9], the use of comics has expanded to include facilitating students in exploring their professional identity, grappling with medical ethics, and comprehending complex health information [10, 11, 12]. Importantly, this evolution has not been confined solely to medical education; recent literature describes the use of comics in nursing education and in the education of other health professionals [14, 15, 16].

Graphic medicine can help promote essential learning and clinical skills. Studies found that students reported better understanding and appreciation of patient's experiences after reading certain comics [12, 13]. Additionally, students reported that creating comics helped improve their communication and clinical skills [12]. In an undergraduate nursing course, the addition of cartooning to a case study presentation resulted in higher post-test scores [16]. A study of residents reported that graphic medicine content added to their knowledge about clinical conditions [13]. Although more research is needed to determine the effect of comics on specific educational domains, there is evidence to conclude that comics are seen as novel and engaging information sources [13, 15, 17].

The approachable nature of graphic medicine also makes it effective for patient care. In the clinical realm, comics have been used for patient education, health literacy, and health promotion. Comic book interventions for preoperative education have been found to reduce anxiety in children and adolescents [18]. Comic books can also facilitate health literacy of complex [19] or stigmatized topics [20]. A study of patients at high risk for metabolic syndrome demonstrated that the addition of educational materials in comic format resulted in increases in healthy eating and physical activity behaviors [21]. Aligning with the findings from educational research, patients have also identified comics as an enjoyable format for learning [19].

As institutions that support health science education, health science libraries adapt and respond to the curricular changes of the programs they support. Health science librarians are poised to identify curricular needs and actualize collection resources. The National Library of Medicine (NLM) has recognized the significant contribution that graphic medicine can have in healthcare, amassing a collection of forty titles by 2018 [22]. Furthermore, NLM curated the exhibit titled *Graphic Medicine: Ill Conceived and Well Drawn* to explore the graphic medicine genre and showcase valuable programming ideas for libraries supporting health science education [22].

Despite this national recognition, a 2020 survey of graphic novels in academic libraries revealed that graphic novels make up less than one percent of academic library collections [23]. Given the increased reporting of graphic medicine usage in medical education, we wanted to see if these statistics also held true for health science library collections. In this study, we performed an analysis of academic health science library catalogs with two primary objectives: to determine which, if any, recommended graphic medicine titles in print format are contained within the collection, and to see which graphic medicine titles could be found most often in these collections. Our secondary objectives were to gauge if library users could access graphic medicine titles, either through other libraries within the same system or as an eBook, and to examine if libraries with graphic medicine titles highlighted these collections on their websites.

## METHODS

We adapted the methodology used by Kevin Mulcahy [24] in “Science Fiction Collections in ARL Academic Libraries” and Cassie Wagner [25] in “Graphic Novel Collections in Academic ARL Libraries” to develop a list of health science libraries and graphic medicine titles. Instead of the Association of Research Libraries member list, we used the Association of Academic Health Science Libraries (AAHSL) member list. This allowed us to focus on libraries that support the information and knowledge needs in the health care environment [26].

We excluded the following to reflect the focus of our study:

1. Libraries that are not solely a health science library
2. Non-academic organizations/libraries
3. Health science libraries (HSL) with catalogs that require an organizational login to search
4. Libraries whose online catalogs do not differentiate holding locations within a library system
5. Libraries without print collections
6. Virtual libraries.

Health science libraries with multiple locations using the same catalog were counted as one entity. The final academic HSL list used for this study was developed between December 2022 and January 2023. It consisted of 125 libraries and is included in Appendix A.

Recommended title and award lists are a staple of library collection development; however, while there are existing lists for graphic novel titles, few are devoted to the subset of graphic medicine. We reviewed graphic novel award lists for titles that were graphic medicine in nature. Additionally, we examined the following graphic medicine award lists and collection development resources for graphic medicine titles:

1. *Essential Graphic Medicine: An Annotated Bibliography*, a list of thirty graphic medicine titles curated in 2018 by health science librarians Alice Jaggers and Matthew Noe [27]. This list was designed as a collection development tool for librarians interested in starting graphic medicine collections.
2. The Graphic Medicine International Collective (GMIC) shortlist for the inaugural 2022 GMIC Award for outstanding health-related comic projects completed and or published in 2021 [28]. The GMIC was founded as a not-for-profit organization in 2019 to support the use of comics in health.
3. The American Library Association's (ALA) Graphic Novels and Comics Round Table's (GNCRT) best graphic novel lists for the years 2020 through 2022. [29]. ALA published the first list in 2020, and all available years were consulted for titles.
4. Award lists from the Eisner Award, Kirkus Prize, Hugo Award, Benjamin Franklin Award, and the Doug Wright Award. No titles from these lists were chosen either because they were not graphic medicine in nature or were duplicated in another list.

The final title list used for this study encompassed all thirty books from Essential Graphic Medicine and twenty that address health-related topics from the ALA's Best Graphic Novels for Adults Reading List and Best Graphic Novels for Children Reading List. Since this project focuses on university-affiliated health sciences libraries, selections from the children's reading list were limited to those that are indicated for older readers. Seven titles from the GMIC Award shortlist were also incorporated. Books that are not readily available for purchase in the U.S., not available in English, or only available in electronic format were excluded. The final list consisted of fifty-seven graphic medicine titles and can be found in Appendix B.

We decided to focus on identifying titles available in print format at the health science library in an attempt to ascertain the collection development priorities of health science libraries. Since electronic resources lack specific holding locations, we felt that eBooks might reflect priorities from other groups within the library system. Furthermore, the availability of individual titles can fluctuate due to purchasing or licensing agreements and may not be connected to a targeted plan. We adopted the following approach to identify print graphic medicine works held within health science library collections. We performed title searches directly in each institution's online catalog to provide the most accurate information about an individual library's holdings. We did not search using either the genre/form terms “Graphic medicine (comics)” or “Graphic Novel(s)” due to inconsistencies in local cataloging practices and the application of genre/form terms.

We developed and tested a standardized data collection worksheet using Microsoft Forms. We collected the following information based on holding information found during the title search:

1. If the title was available in print format at the health science library location. This included books that were noted as lost, on order, or on reserve.
2. If the print book was available from another (non-health specific) library in the system
3. If the title was available as an eBook
4. If the library website featured a graphic medicine collection
5. If the library website had a library guide on graphic medicine.

Data was collected from April to August 2023. Additional content information was collected from the identified library guides in February 2024.

## RESULTS

### Q1: Are graphic medicine titles available in health science libraries?

**Figure 2:**
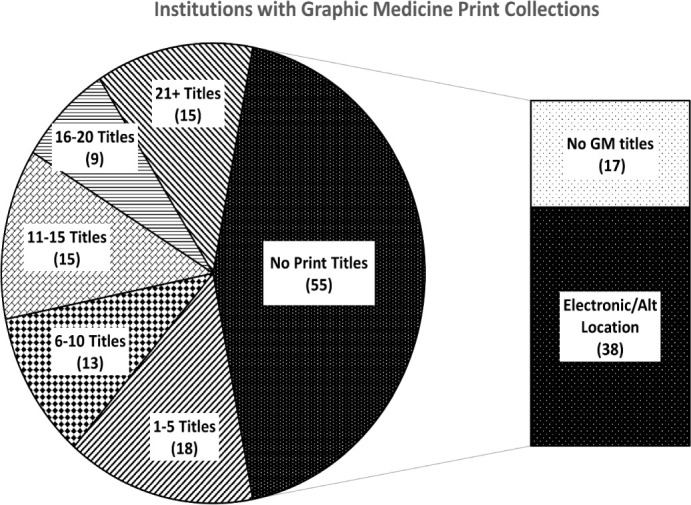
Health Science Libraries with Graphic Medicine Titles

Of the 125 library catalogs examined, seventy had at least one graphic medicine title from the list in their print collection. This represents fifty-six percent of the total libraries analyzed (Figure 2). Twelve percent of libraries had larger collections of twenty-one titles or more.

### Q2. Are graphic medicine titles available as eBooks or through other libraries within the university's library system?

Figure 2 also shows the prevalence of graphic medicine titles available as eBooks or from other libraries at the university among those with no print graphic medicine titles. Of the fifty-five libraries that did not have any print titles in their collections, thirty-eight had access to the titles as an eBook. Seventy percent of all library catalogs examined had access to at least one graphic medicine title on the list as an eBook. Additionally, sixty-two percent had access to print titles through other libraries at their university. Of all the libraries examined only seventeen (14%) had no access to any of the graphic medicine titles on the list through either a print collection, eBooks, or other libraries at their university.

### Q3: What graphic medicine titles are most often found in health science library collections?

Titles from the Essential Graphic Medicine list featured more prominently in library collections compared to titles from other resources we consulted. The Essential Graphic Medicine list represented a little over fifty percent of the complete list developed for this project and included the earliest titles dating from 2006 to 2019. The other sources used to develop the graphic medicine title list only included titles published from 2020 to 2022. A complete list of titles and their presence in library collections is in Appendix C.

### Q4: Do libraries feature graphic medicine library guides or collection information on their websites?

**Figure 3:**
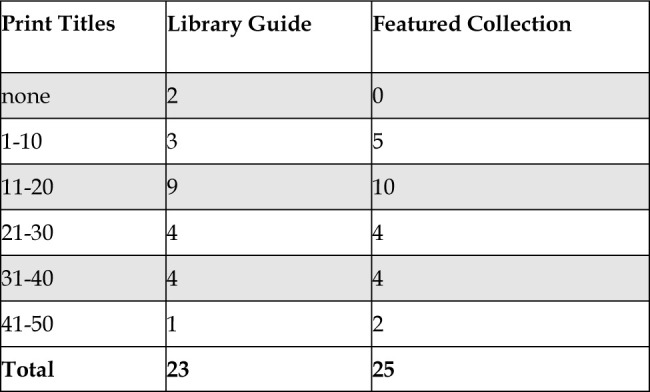
Total library guides and feature collections by print collection size.

Figure 3 shows the number of library guides and featured collections in relation to the size of the print collections. Of the academic health science library websites examined, twenty-five had graphic medicine collection information on their website and/or on a library guide related to graphic medicine. This information was in the form of individual books with catalog information or permalinks to the collection in the catalog. Approximately a third of the institutions with print titles from our list had a library guide (21) and/or featured collection (25). Two libraries with no print titles had a library guide.

## DISCUSSION

Among the health science libraries analyzed, over half included graphic medicine titles from our list in their print collections. However, roughly a quarter of these had fewer than ten titles. This is not a surprising finding given the trend in academic libraries to reduce print collections in favor of electronic resources [30]. Our data confirmed this trend: 70% of libraries offer at least one graphic medicine title in eBook format compared to only 56% with print copies. Most of the libraries without print editions had access to the titles in electronic format (69%).

Although a detailed discussion of acquisition decisions and the reasons why a library may acquire a title in a specific format is outside the scope of this study, based upon our data it seems likely that health science libraries may not be collecting graphic medicine titles in print if other libraries within their university offer them, or if they are available as eBooks. Academic libraries are generally on tight budgets and may be putting their funds toward resources that are more readily accessible (eBooks) or may have collaborative collection development practices in place that discourage duplicating materials that are present within their overall system. Additionally, health science libraries may face collection development constraints, such as requirements to purchase from specific vendors, prioritize curriculum-related materials, or acquire electronic formats due to space limitations. Although the eBook format was found to be more prevalent in the academic libraries examined, a study of undergraduate and graduate students found that the preferred format for comics is print [31]. Librarians investing in graphic medicine materials may consider this preference when adding to or developing their collections.

Titles from the Essential Graphic Medicine list were most frequently found in library collections. Essential Graphic Medicine is one of the few collection development resources available for this genre and may be influencing acquisition choices. This is evident in the title list developed for this study as slightly over fifty percent of our entire list came from this source and may have skewed the results in favor of finding these titles. Additionally, the Essential Graphic Medicine list includes older titles dating back to 2006 allowing libraries more time to add them to their collection. Considering that graphic medicine is a relatively young genre, it's unsurprising that some libraries lack titles in this category.

We hypothesized that the existence of a graphic medicine library guide would be associated with the size of a health science library's graphic medicine collections. Among the twenty-three library guides observed, eighteen originated from libraries with twelve or more graphic medicine titles. Conversely, only five library guides were associated with libraries possessing fewer than ten titles. Further examination revealed these libraries instead had electronic collections of graphic medicine titles. Most libraries with graphic medicine library guides (twenty-two of twenty-three) provided information specifically about the graphic medicine collection within the library. This aligns with the common practice of featuring collection resources in library guides [32]. Although many library guides featured collection information, we can draw no direct correlation between the size of the collection and the existence of a library guide. The presence of a library guide may be driven by other factors like a librarian's interest in graphic medicine or course offerings on or using graphic medicine.

Librarians with graphic medicine collections are actively promoting graphic medicine as a topic, both as a teaching tool and for research purposes on their library guides. Eighteen of the libraries with library guides included topical information and links to relevant resources. Research-oriented content, such as scholarly articles and database search strings, were featured in eleven guides. Additionally, nine of the library guides emphasized teaching with graphic medicine, offering examples, assignments, syllabi, and tools for creating comics. Notably, one guide was dedicated entirely to a graphic medicine course. Given its value in traditional medical education [9], graphic medicine occupies a unique position. Librarians play a pivotal role in advocating for its use both as a research resource and as an educational aid. By showcasing resources that demonstrate graphic medicine's application in research and teaching, libraries can drive utilization of existing collections and contribute to the advancement of this genre.

## LIMITATIONS/FUTURE DIRECTIONS

Our decision to use a selected title list and to focus on print titles limited the scope of our findings. Institutions may hold additional titles not included in our list. We discovered at least two libraries with other graphic medicine titles available in their collections. Some libraries do not have print collections or are no longer investing in print materials. While we documented cases where electronic editions were present in the catalog, we did not include these in our primary study for reasons described in our methodology.

Focusing solely on academic health science libraries does not take into consideration general academic libraries that support health professional programs or collaborative collection development practices that may exist amongst university library systems. A survey of graphic novel collections in Association of Research Libraries (ARL) institutions found that although they tend to be small, graphic novel collections are often present in ARL libraries [25]. General libraries may be collecting graphic medicine titles that support multiple programs offered at their universities.

Future studies could address these limitations by exploring the resources that librarians consult for collection development, investigating specific titles available through the eBook format, and titles recommended for younger age ranges since all the titles on our list are indicated for fifth grade and above. Additionally, the ALA's Graphic Novels and Comics Round Table's “Best Practices for Cataloging Comics and Graphic Novels Using RDA and MARC21” published in 2022 [33] may help to standardize and align cataloging practices, allowing for more targeted and efficient search methods to be employed in future studies.

Examining the collections of all academic libraries that support health professional programs could provide further insight into the prevalence of graphic medicine collections along with factors that may be driving collection development such as librarian interest, budget, and program needs. Additionally, circulation statistics may add information about the preference between print and electronic titles and how these materials are used in educational programs outside of health.

## CONCLUSIONS

Graphic medicine is a relatively young genre with applications across health science professional programs. This study provides a starting point for describing the prevalence and breadth of graphic medicine collections in academic health science libraries. Only fourteen percent of libraries in this study did not have any graphic medicine titles available in either print or electronic editions. This suggests that, although their presence may be small, academic health science libraries are collecting graphic medicine titles. Additionally, the use of graphic medicine in both research and education is an emerging field [6]. Academic librarians can support the growing interest in the comic art format by incorporating graphic medicine titles into their collections and educating their patrons on this important genre.

## Data Availability

Data associated with this article are available in the Open Science Framework at https://doi.org/10.17605/OSF.IO/NBMWX
