# Supplementary material for: Graphic medicine in academic health science library collections
Source: J Med Libr Assoc. 2025 Aug 1;113(3):233–40. doi: 10.5195/jmla.2025.1962 (PMC12369966; doi:10.5195/jmla.2025.1962)
Supplement: Supplementary file 1 — Appendix A [file jmla-113-3-233-s01.pdf]

Appendix A. Academic Health Science Libraries

Institution	Library
Boston University Medical Campus	Alumni Medical Library
Vanderbilt University	Annette and Irwin Eskind Family Biomedical Library and Learning Center
University of Kansas Medical Center	AR Dykes Library
A T Still University of Health Sciences	AT Still Memorial Library
Columbia University Irving Medical Center	Augustus C Long Health Sciences Library
Washington University School of Medicine in St Louis	Bernard Becker Medical Library
Charles R Drew University of Medicine and Science	CDU Health Sciences Library
University of South Alabama	Charles M Baugh Biomedical Library
University of South Carolina	Charles S and Donna H Bryan School of Medicine Library
Florida State University	Charlotte Edwards Maguire Medical Library
California Health Sciences University	CHSU Health Sciences Library
University of Virginia	Claude Moore Health Sciences Library
Case Western Reserve University	Cleveland Health Sciences Library
Rowan University	Cooper Medical School of Rowan University Library and Rowan-Virtua SOM Health Sciences Library
Wake Forest University School of Medicine	Coy C Carpenter Library
Albert Einstein College of Medicine	D Samuel Gottesman Library
Georgetown University	Dahlgren Memorial Library
University of Vermont	Dana Medical Library
Loma Linda University	Del E Webb Memorial Library
University of Texas Health Science Center San Antonio	Dolph Briscoe Jr Library
University of Cincinnati	Donald C Harrison Health Sciences Library
University of Wisconsin-Madison	Ebling Library for the Health Sciences
Quinnipiac University	Edward and Barbara Netter Library
Eastern Virginia Medical School	Edward E Brickell Medical Sciences Library
University of Rochester Medical Center	Edward G Miner Library
University of California – Davis	F William Blaisdell MD Medical Library (Blaisdell Medical Library)
University of California – Irvine	Forest J Grunigen Medical Library (GML)
Harvard Medical School	Francis A Countway Library of Medicine
Northwestern University Feinberg School of Medicine	Galter Health Sciences Library & Learning Center

University of North Texas Health Science Center	Gibson D Lewis Health Science Library
University of Iowa	Hardin Library for the Health Sciences
Penn State College of Medicine	Harrell Health Sciences Library Research and Learning Commons
University of Central Florida	Harriet F Ginsburg Health Sciences Library
Yale University	Harvey Cushing John Hay Whitney Medical Library
University of the Incarnate Word	Health Professions Libraries
Marian University	Health Professions Library
Dartmouth College	Health Sciences and Biomedical Libraries
University of Maryland, Baltimore	Health Sciences and Human Services Library
University at Buffalo	Health Sciences at Abbott Library
University of Minnesota – Twin Cities	Health Sciences Libraries
Creighton University	Health Sciences Library
Donald and Barbara Zucker School of Medicine at Hofstra/Northwell	Health Sciences Library
Loyola University Chicago	Health Sciences Library
Massachusetts College of Pharmacy and Health Sciences	Health Sciences Library
Stony Brook University	Health Sciences Library
SUNY Upstate Medical University	Health Sciences Library
The Ohio State University	Health Sciences Library
The University of Tennessee Health Science Center	Health Sciences Library
University of Houston	Health Sciences Library
University of Arizona	Health Sciences Library - Tucson Health Sciences Library - Phoenix
University of New Mexico	Health Sciences Library & Informatics Center
University of Hawai'i at Mānoa	Health Sciences Library John A Burns School of Medicine
University of Pittsburgh	Health Sciences Library System – Falk Library
George Washington University	Himmelfarb Health Sciences Library
Tufts University	Hirsh Health Sciences Library
Idaho College of Osteopathic Medicine	ICOM Medical Library
Louisiana State University Health – New Orleans & Shreveport	John P Isché Library LSU Health Shreveport Library
Kansas City University	KCU Libraries
University of Massachusetts Chan Medical School	Lamar Soutter Library
Stanford Medicine	Lane Medical Library
East Carolina University	Laupus Health Sciences Library
University of Nebraska Medical Center	Leon S McGoogan Health Sciences Library

Roseman University of Health Sciences	Library
Rush University Medical Center	Library of Rush University Medical Center
The University of Illinois at Chicago	Library of the Health Sciences
University of Alabama at Birmingham	Lister Hill Library of the Health Sciences
University of Miami Miller School of Medicine	Louis Calder Memorial Library
Howard University	Louis Stokes Health Sciences Library
University of California – Los Angeles	Louise M Darling Biomedical Library
Morehouse School of Medicine	M Delmar Edwards MD Library
Nova Southeastern University	Martin & Gail Press Health Professions Division Library
Medical College of Wisconsin	MCW Libraries
Saint Louis University	Medical Center Library
University of Kentucky	Medical Center Library
Duke University	Medical Center Library & Archives
Florida International University	Medical Library
Oklahoma State University Center for Health Sciences	Medical Library
Southern Illinois University School of Medicine	Medical Library
Texas A&M University	Medical Sciences Library
Midwestern University	Midwestern University Library
University of Texas Medical Branch at Galveston	Moody Medical Library
University of Toledo	Mulford Health Science Library
Medical University of South Carolina	MUSC Libraries
University of Southern California	Norris Medical Library
NYU Langone Health	NYU Health Sciences Library
Oregon Health and Science University	OHSU Library
Philadelphia College of Osteopathic Medicine	OJ Snyder Memorial Library
The University of Tennessee Graduate School of Medicine	Preston Medical Library
Augusta University	Robert B Greenblatt MD Library
University of Oklahoma Health Sciences Center	Robert M Bird Health Sciences Library
Rutgers – New Brunswick	Robert Wood Johnson Library of the Health Sciences
Boxer Library	Rosalind Franklin University of Medicine and Science
University of Mississippi Medical Center	Rowland Medical Library
Tulane University	Rudolph Matas Library of the Health Sciences
Indiana University School of Medicine	Ruth Lily Medical Library
Weill Cornell Medicine	Samuel J Wood Library

University of Nevada, Reno School of Medicine	Savitt Medical Library
Albany Medical College	Schaffer Library of Health Sciences
University of Nevada – Las Vegas	School of Medicine Library
Thomas Jefferson University	Scott Memorial Library
Temple University	Simmy and Harry Ginsburg Health Sciences Library
Mercer University	Skelton Medical Libraries
University of North Dakota – School of Medicine & Health Sciences	SMHS Library Resources
University of Utah	Spencer S Eccles Health Sciences Library
Washington State University	Spokane Academic Library
University of Pennsylvania	STEM Libraries – Holman Biotech Commons
University of Colorado Anschutz Medical Campus	Strauss Health Sciences Library
University of Michigan	Taubman Health Sciences Library
Arkansas Colleges of Health Education	Taylor Health Sciences Library
Seton Hall University	The Interprofessional Health Sciences Library (IHS Library)
The University of Chicago	The John Crerar Library
Texas Tech University Health Sciences Center El Paso	TTUHSC El Paso Libraries
Texas Tech University Health Sciences Center	TTUHSC Libraries of the Health Sciences
University of Arkansas for Medical Sciences	UAMS Library
UConn Health	UConn Health Sciences Library
University of California – San Francisco	UCSF Library
University of Florida	UF Health Science Center Libraries
University of South Florida	USF Health Libraries
University of Texas Rio Grande Valley	UTRGV School of Medicine Libraries
University of Washington	UW Health Sciences Library
Virginia Commonwealth University	VCU Health Sciences Library
Wayne State University	Vera P Shiffman Medical Library
University of South Dakota	Wegner Health Sciences Library
Johns Hopkins University	William H Welch Medical Library
Emory University	Woodruff Health Sciences Center Library
