# Supplementary material for: Graphic medicine in academic health science library collections
Source: J Med Libr Assoc. 2025 Aug 1;113(3):233–40. doi: 10.5195/jmla.2025.1962 (PMC12369966; doi:10.5195/jmla.2025.1962)
Supplement: Supplementary file 2 — Appendix B [file jmla-113-3-233-s02.pdf]

Appendix B. Graphic Medicine Book Titles

Title	Author
A-Okay	Jarad Greene
Living with Viola	Rosena Fung
Smaller Sister	Maggie Edkins Willis
Stone Fruit	Lee Lai
Little Josephine: Memory in Pieces	Valérie Villieu
The CL Psychiatrist	Omar Mirza
Wash day diaries	Jamila Rowser
Fine: A Comic About Gender.	Rhea Ewing
Resistance	VAl McDermid
Ripple Effects	Jordan Hart
Everything is OK	Debbie Tung
My Life in Transition: A Super Late Bloomer Collection	Julie Kaye
Spellbound	Bishakh Som
The Most Costly Journey: Stories of Migrant Farmworkers in Vermont Drawn by New England Cartoonists	Marek Bennett
INvisible Differences	Julie Dachez
My Alcoholic Escape from Reality	Nagata Kabi
The Golden Hour	Niki Smith
Coma	Zara Slattery
Down to the Bone: A Leukemia Story	Catherine Pioli
SENSORY: Life on the Spectrum: An Autistic Comics Anthology	Rebessa Ollerton
Catalogue Baby: A Memoir of (In)fertility.	Myriam Steinberg
The Magic Fish	Trung Le Nguyen
The Secret to Superhuman Strength	Alison Bechdel
Two Week Wait: An IVF Story	Luke and Kelly Jackson
Becoming Unbecoming	Una
Kimiko Does Cancer: A Graphic Memoir	Kimiko Tobimatsu
Lissa: A Story About Medical Promise, Friendship, and Revolution	Sherine Hamdy, Coleman Nye
The Body Factory: From the First Prosthetics to the Augmented Human	Héloïse Chochois
Parenthesis	Élodie Durand.
Dumb: Living Without A Voice	Georgia Webber
The Infinite Wait and Other Stories	Julia Wertz
Billy, Me & You: A Memoir of Grief and Recovery	Nicola Streeten
Wrinkles (Arrugas)	Paco Roca

Mis(h)adra	Iasmin Omar Ata
El Deafo	Cece Bell
Smile	Raina Telgemeier
Hey, Kiddo: How I Lost My Mother, Found My Father, and Dealt with Family Addiction	Jarrett J. Krosoczka
Hyperbole and a Half: Unfortunate Situations, Flawed Coping Mechanisms, Mayhem, and Other Things That Happened	Allie Brosh
Rosalie Lightning	Tom Hart
Kid Gloves: Nine Months of Careful Chaos	Lucy Knisley
Gender Queer: A Memoir	Maia Kobabe
The Facts of Life	Paula Knight
Hole in the Heart: Bringing Up Beth	Henny Beaumont
Cancer Made Me a Shallower Person	Miriam Engelberg
Lighter Than My Shadow	Katie Green
Mom's Cancer	Brian Fies
Stitches	David Small
My Degeneration: A Journey Through Parkinson's	Peter Dunlap-Shohl
Aliceheimer's: Alzheimer's Through the Looking Glass	Dana Walrath
Cancer Vixen: A True Story	Marisa Acocella
Rx: A Graphic Memoir	Rachel Lindsay
Tangles: A Story About Alzheimer's, My Mother, and Me	Sarah Leavitt
Can't We Talk About Something More Pleasant?: A Memoir	Roz Chast
Marbles: Mania, Depression, Michelangelo, and Me	Ellen Forney
Graphic Medicine Manifesto	MK Czerwiec, et al
The Bad Doctor: The Troubled Life and Times of Dr. Iwan James	Ian Williams
Taking Turns: Stories from HIV/AIDS Care Unit 371	MK Czerwiec
