# Supplementary material for: Graphic medicine in academic health science library collections
Source: J Med Libr Assoc. 2025 Aug 1;113(3):233–40. doi: 10.5195/jmla.2025.1962 (PMC12369966; doi:10.5195/jmla.2025.1962)
Supplement: Supplementary file 3 — Appendix C [file jmla-113-3-233-s03.docx]

| **RANK** | **TITLE** | **AUTHOR** | **YEAR** | **HSLs*** |
| --- | --- | --- | --- | --- |
| 1 | Taking Turns: Stories from HIV/AIDS Care Unit 371 | MK Czerwiec | 2017 | 49 |
| 2 | The Bad Doctor: The Troubled Life and Times of Dr. Iwan James | Ian Williams | 2014 | 47 |
| 3 | Graphic Medicine Manifesto | MK Czerwiec, et al | 2015 | 41 |
| 4 | Can’t We Talk About Something More Pleasant?: A Memoir | Roz Chast | 2014 | 38 |
| 4 | Marbles: Mania, Depression, Michelangelo, And Me | Ellen Forney | 2012 | 38 |
| 6 | Tangles: A Story About Alzheimer’s, My Mother, and Me | Sarah Leavitt | 2012 | 36 |
| 7 | Aliceheimer’s: Alzheimer’s Through the Looking Glass | Dana Walrath | 2016 | 35 |
| 7 | Cancer Vixen: A True Story | Marisa Acocella | 2006 | 35 |
| 7 | Rx: A Graphic Memoir | Rachel Lindsay | 2018 | 35 |
| 10 | My Degeneration: A Journey Through Parkinson’s | Peter Dunlap-Shohl | 2015 | 34 |
| 11 | Stitches | David Small | 2009 | 31 |
| 12 | Mom’s Cancer | Brian Fies | 2006 | 30 |
| 13 | Cancer Made Me a Shallower Person | Miriam Engelberg | 2006 | 29 |
| 13 | Lighter Than My Shadow | Katie Green | 2013 | 29 |
| 15 | Hole in the Heart: Bringing Up Beth | Henny Beaumont | 2016 | 28 |
| 16 | Gender Queer: A Memoir | Maia Kobabe | 2019 | 26 |
| 16 | The Facts of Life | Paula Knight | 2017 | 26 |
| 18 | Kid Gloves: Nine Months of Careful Chaos | Lucy Knisley | 2019 | 24 |
| 19 | Hyperbole and a Half: Unfortunate Situations, Flawed Coping Mechanisms, Mayhem, and Other Things That Happened | Allie Brosh | 2013 | 23 |
| 19 | Rosalie Lightning | Tom Hart | 2016 | 23 |
| 21 | Hey, Kiddo: How I Lost My Mother, Found My Father, and Dealt with Family Addiction | Jarrett J. Krosoczka | 2018 | 20 |
| 22 | El Deafo | Cece Bell | 2014 | 18 |
| 22 | Smile | Raina Telgemeier | 2010 | 18 |
| 24 | Mis(h)adra | Iasmin Omar Ata | 2017 | 17 |
| 25 | Billy, Me & You: A Memoir of Grief and Recovery | Nicola Streeten | 2011 | 16 |
| 25 | Wrinkles (Arrugas) | Paco Roca | 2016 | 16 |
| 27 | Dumb: Living Without a Voice | Georgia Webber | 2018 | 15 |
| 27 | The Infinite Wait and Other Stories | Julia Wertz | 2012 | 15 |
| 29 | Parenthesis | Élodie Durand. | 2021 | 14 |
| 30 | The Body Factory: From the First Prosthetics to the Augmented Human | Héloïse Chochois | 2021 | 13 |
| 31 | Becoming Unbecoming | Una | 2015 | 12 |
| 31 | Kimiko Does Cancer: A Graphic Memoir | Kimiko Tobimatsu | 2020 | 12 |
| 31 | Lissa: A Story About Medical Promise, Friendship, and Revolution | Sherine Hamdy, Coleman Nye | 2017 | 12 |
| 34 | Two Week Wait: An IVF Story | Luke and Kelly Jackson | 2021 | 9 |
| 35 | Catalogue Baby: A Memoir of (In)fertility. | Myriam Steinberg | 2021 | 8 |
| 35 | The Magic Fish | Trung Le Nguyen | 2020 | 8 |
| 35 | The Secret to Superhuman Strength | Alison Bechdel | 2021 | 8 |
| 38 | Coma | Zara Slattery | 2021 | 7 |
| 38 | Down to the Bone: A Leukemia Story | Catherine Pioli | 2022 | 7 |
| 38 | SENSORY: Life on the Spectrum: An Autistic Comics Anthology | Rebessa Ollerton | 2022 | 7 |
| 41 | INvisible Differences | Julie Dachez | 2020 | 6 |
| 41 | My Alcoholic Escape from Reality | Nagata Kabi | 2021 | 6 |
| 41 | The Golden Hour | Niki Smith | 2021 | 6 |
| 44 | Everything is OK | Debbie Tung | 2022 | 5 |
| 44 | My Life in Transition: A Super Late Bloomer Collection | Julie Kaye | 2021 | 5 |
| 44 | Spellbound | Bishakh Som | 2020 | 5 |
| 44 | The Most Costly Journey: Stories of Migrant Farmworkers in Vermont Drawn by New England Cartoonists | Marek Bennett | 2021 | 5 |
| 48 | Fine: A Comic About Gender. | Rhea Ewing | 2022 | 4 |
| 48 | Resistance | VAl McDermid | 2021 | 4 |
| 48 | Ripple Effects | Jordan Hart | 2022 | 4 |
| 51 | Little Josephine: Memory in Pieces | Valérie Villieu | 2020 | 3 |
| 51 | The CL Psychiatrist | Omar Mirza | 2021 | 3 |
| 51 | Wash Day Diaries | Jamila Rowser | 2022 | 3 |
| 54 | Stone Fruit | Lee Lai | 2021 | 2 |
| 55 | Living with Viola | Rosena Fung | 2021 | 1 |
| 55 | Smaller Sister | Maggie Edkins Willis | 2022 | 1 |
| 57 | A-Okay | Jarad Greene | 2021 | 0 |
